# Plaque Wall Distribution Pattern of the Atherosclerotic Middle Cerebral Artery Associates With the Circle of Willis Completeness

**DOI:** 10.3389/fneur.2020.599459

**Published:** 2021-01-11

**Authors:** Jia Li, Lu Zheng, Wen-Jie Yang, Cheuk-Yin Sze-To, Thomas Wai-Hong Leung, Xiang-Yan Chen

**Affiliations:** ^1^Department of Health Technology and Informatics, The Hong Kong Polytechnic University, Kowloon, Hong Kong; ^2^Department of Neurology, The Third Affiliated Hospital of Sun Yat-sen University, Guangzhou, China; ^3^The Russell H. Morgan Department of Radiology and Radiological Sciences, The Johns Hopkins Hospital, Baltimore, MD, United States; ^4^Department of Diagnostic and Interventional Radiology, The Hong Kong Sanatorium & Hospital, Hong Kong, Hong Kong; ^5^Division of Neurology, Department of Medicine and Therapeutics, The Prince of Wales Hospital, The Chinese University of Hong Kong, Shatin, Hong Kong

**Keywords:** intracranial atherosclerosis (ICAS), middle cerebral artery (MCA), plaque wall distribution, circle of Willis (CoW), vascular anatomical variation

## Abstract

**Objective:** Investigating the relevance of the incomplete circle of Willis (COW) to the plaque wall distribution in the atherosclerotic middle cerebral arteries (MCAs) through utilizing high-resolution magnetic resonance imaging (HR-MRI), and its potential clinical impact.

**Methods:** This hospital-based study enrolled consecutive adult patients with acute ischemic stroke or transient ischemic attack, who received a 3.0T Achieva MR system scanning. The COW completeness was evaluated on MR angiography imaging, including anterior (A) and posterior (P)-COW sections. The MCA plaque wall distribution was assessed on HR-MRI. The occurrence of perforator infarction was detected on diffusion-weighted imaging.

**Results:** Among 87 patients (mean age = 62.39 ± 11.64 years old) with atherosclerotic plaques in the MCA M1 segments, the incomplete COW types were more prevalent than the complete COW type (incomplete P-COW, 83.9%; incomplete A-COW, 36.8%; complete COW, 8.1%). The incomplete A-COW had more inferior but fewer ventral plaques of MCA atherosclerosis than the complete A-COW, while the incomplete P-COW had fewer inferior MCA plaques than the complete P-COW. Moreover, symptomatic MCA plaques causing perforator infarctions were more likely to locate on the superior wall.

**Conclusion:** Our findings suggested that the COW completeness could influence the vessel wall distribution of the MCA plaques, among which the superior plaques of symptomatic MCA atherosclerosis was associated with branch occlusive disease.

## Introduction

Intracranial atherosclerosis (ICAS) is the main etiological factor of ischemic stroke (IS) (1), the neurological burden of which remains a threat to the public health worldwide (2, 3). The middle cerebral arteries (MCAs) are the most vulnerable to atherosclerosis (1), with severer luminal narrowing degree and more eccentric plaques (4). Generally, the formation and progression of MCA atherosclerosis are influenced by the geometric variations in the cerebral vascular system (5, 6). The circle of Willis (COW) varies considerably in the anatomical structures among the normal populations (7–9), and may play a decisive role in the regulation of developing ICAS (10, 11). The scarcity of the established relationship between the COW structural completeness and the MCA atherosclerosis features may imply that new evidence is needed to clarify the fundamental mechanisms of ICAS.

High-resolution magnetic resonance imaging (HR-MRI) can serve as a non-invasive vessel wall imaging technique in delineating the direct morphology of intracranial atherosclerotic plaques (12, 13). Describing the essential pattern of the MCA plaque wall distribution via HR-MRI is of clinical importance in exploring the vascular pathology of MCA atherosclerosis and precisely performing the cerebrovascular interventions (5, 14). In the current study, we aimed to examine the association of the incomplete COW with the plaque wall distribution in the atherosclerotic MCAs by using HR-MRI, as well as its underlying clinical impact.

## Materials and Methods

### Study Population

**Figure 1:**
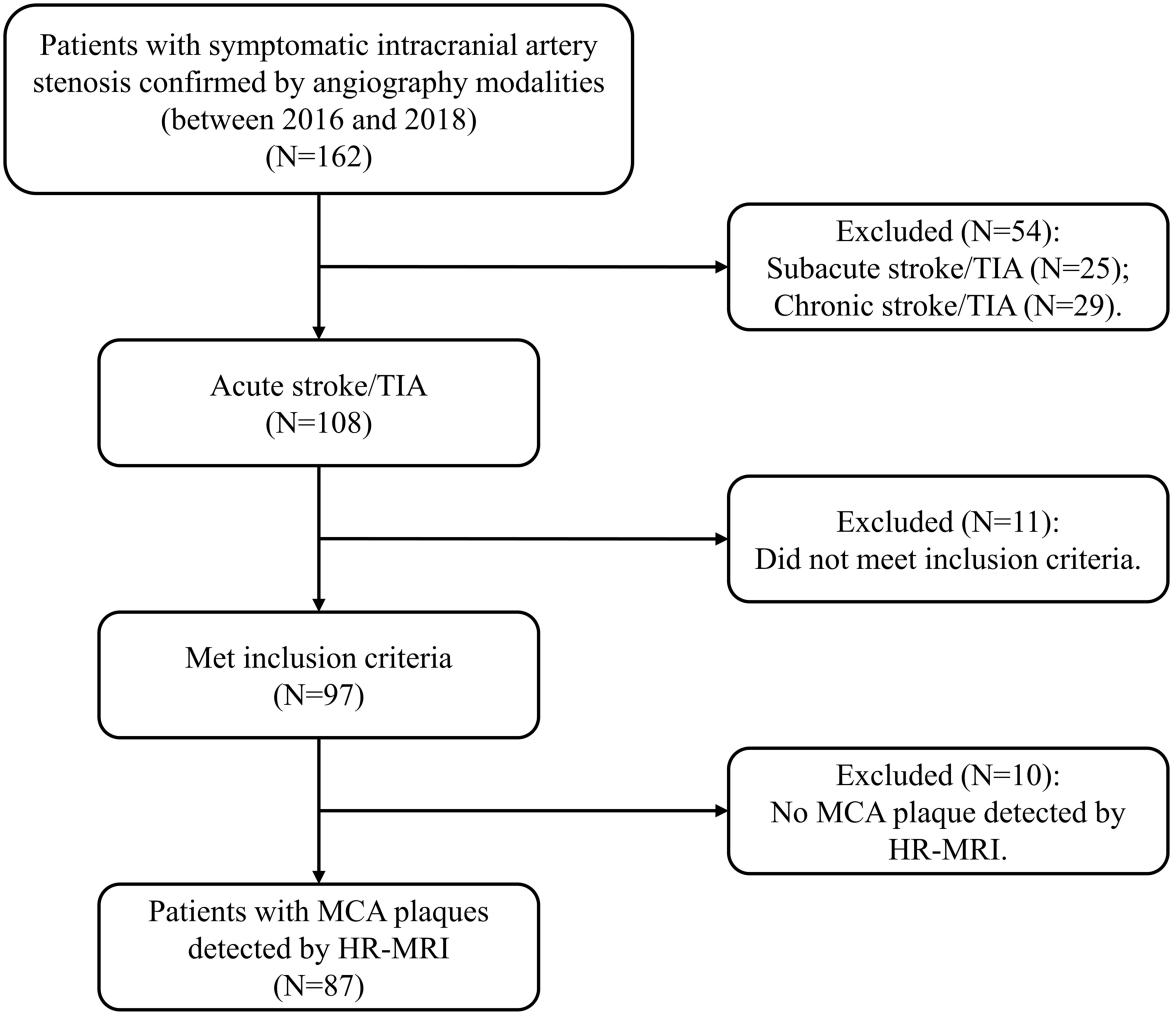
The flow chart indicates the selection of the subjects. HR-MRI, high-resolution magnetic resonance imaging; MCA, middle cerebral artery; TIA, transient ischemic attack.

In this study, adult subjects were consecutively recruited from our single institution (2016–2018). The patients were included, if they met the following criteria: (1) first-ever acute IS or transient ischemic attack (TIA) within 7 days; (2) any intracranial large-artery atherosclerotic stenosis confirmed by magnetic resonance angiography (MRA) or digital subtraction angiography (DSA); (3) MCA atherosclerotic plaques detected by HR-MRI; (4) at least one atherosclerotic risk factor, including hypertension, hyperlipidemia, diabetes and smoking; and (5) good image quality for detecting plaques. The subjects were excluded, if they had any evidence of (1) non-atherosclerotic stenosis (moyamoya disease, vasculitis, or dissection); (2) cardioembolism (valvular heart disease or atrial fibrillation); (3) coexistent moderate to severe internal carotid artery (ICA) or extracranial carotid artery (ECA) stenosis; (4) no MCA atherosclerotic plaque detected by HR-MRI, and (5) contraindications to MRI. The flow chart for the subject inclusion was presented in Figure 1. The institutional review board approved this study which followed the Declaration of Helsinki. All participants provided the written informed consent.

### Imaging Protocol

The imaging protocols of both 3D Time-Of-Flight (TOF) MRA and transverse 3D T1-weighted (T1w) Volumetric ISotropically Turbo spin echo Acquisition (VISTA) sequences were described in our previous MRI study (15).

A 3T Achieva MR system with an 8-channel head coil (Philips Healthcare, Cleveland, OH, USA) was used in this study. All the subjects were scanned by the transverse 3D T1w VISTA sequence before and after the administration of a gadolinium-containing contrast agent (Dotarem, Gadoteric acid 0.5 mmol/mL; Guerbet, Roissy CdG Cedex, France) (0.1 mL/kg to each subject). The interval between the two scans was ~5 min. The T1w VISTA sequence parameters included field-of-view (FOV) 200 × 167 × 45 mm^3^, acquired resolution 0.6 × 0.6 × 1.0 mm^3^, reconstructed resolution 0.5 × 0.5 × 0.5 mm^3^ utilizing zero filling, repetition time (TR) 1,500 ms, echo time (TE) 36 ms, SENSE factor 1.5 (phase-encode direction), echo spacing 4.0 ms, TSE + startup echoes 56 + 6, and scan duration 6:51 min. The 3D TOF MRA sequence was also performed, the parameters of which included FOV 200 × 200 × 56 mm^3^, acquired resolution 0.4 × 0.6 × 0.7 mm^3^, TR/TE 23/3.5 ms, and scan duration 3:07 min.

### Imaging Analyses

**Figure 2:**
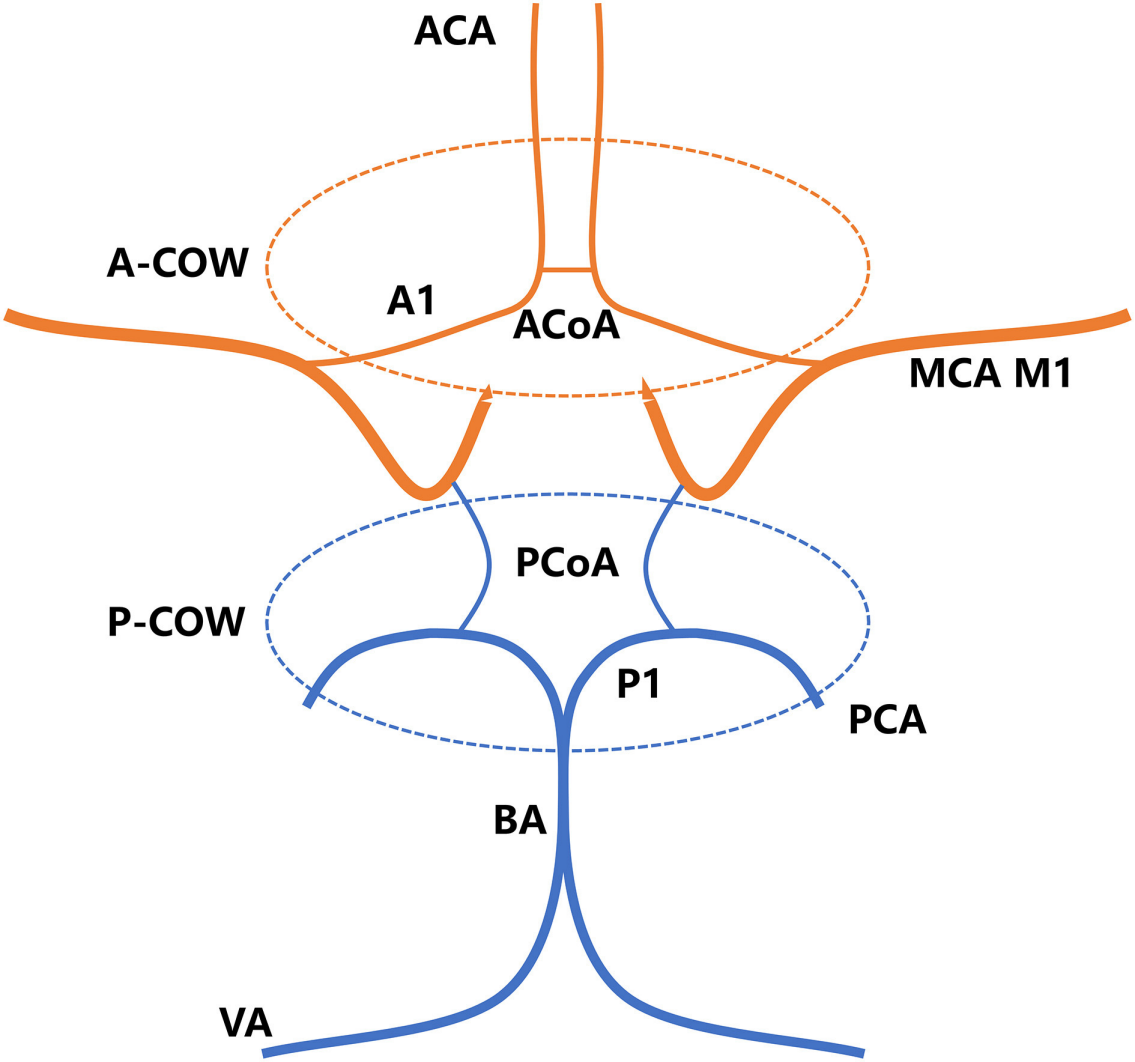
The COW consists of A-COW and P-COW sections. A-COW in red: bilateral A1 segments of ACA and ACoA. P-COW in blue: bilateral P1 segments of PCA and bilateral PCoAs. ACA, anterior cerebral artery; ACoA, anterior communicating artery; BA, basilar artery; COW, circle of Willis; A-COW, anterior COW; P-COW, posterior COW; MCA, middle cerebral artery; PCA, posterior cerebral artery; PCoA, posterior communicating artery; VA, vertebral artery.

Two experienced readers blind to the clinical parameters identified the structural integrity of the COW, according to the findings on MRA (16, 17). The COW comprised two anatomical parts, including anterior COW (A-COW) and posterior COW (P-COW) (Figure 2). An incomplete COW was determined by any absence or dysplasia of vascular structure within each of the two sections, and then classified as incomplete A-COW and incomplete P-COW. If left or right posterior communicating artery was found absent or dysplastic on the MRA images, for instance, the P-COW part was regarded as incomplete. A complete COW was defined by the existence of both complete A-COW and complete P-COW. This image analysis was independent of the subsequent assessment of the MCA plaque wall distribution.

**Figure 3:**
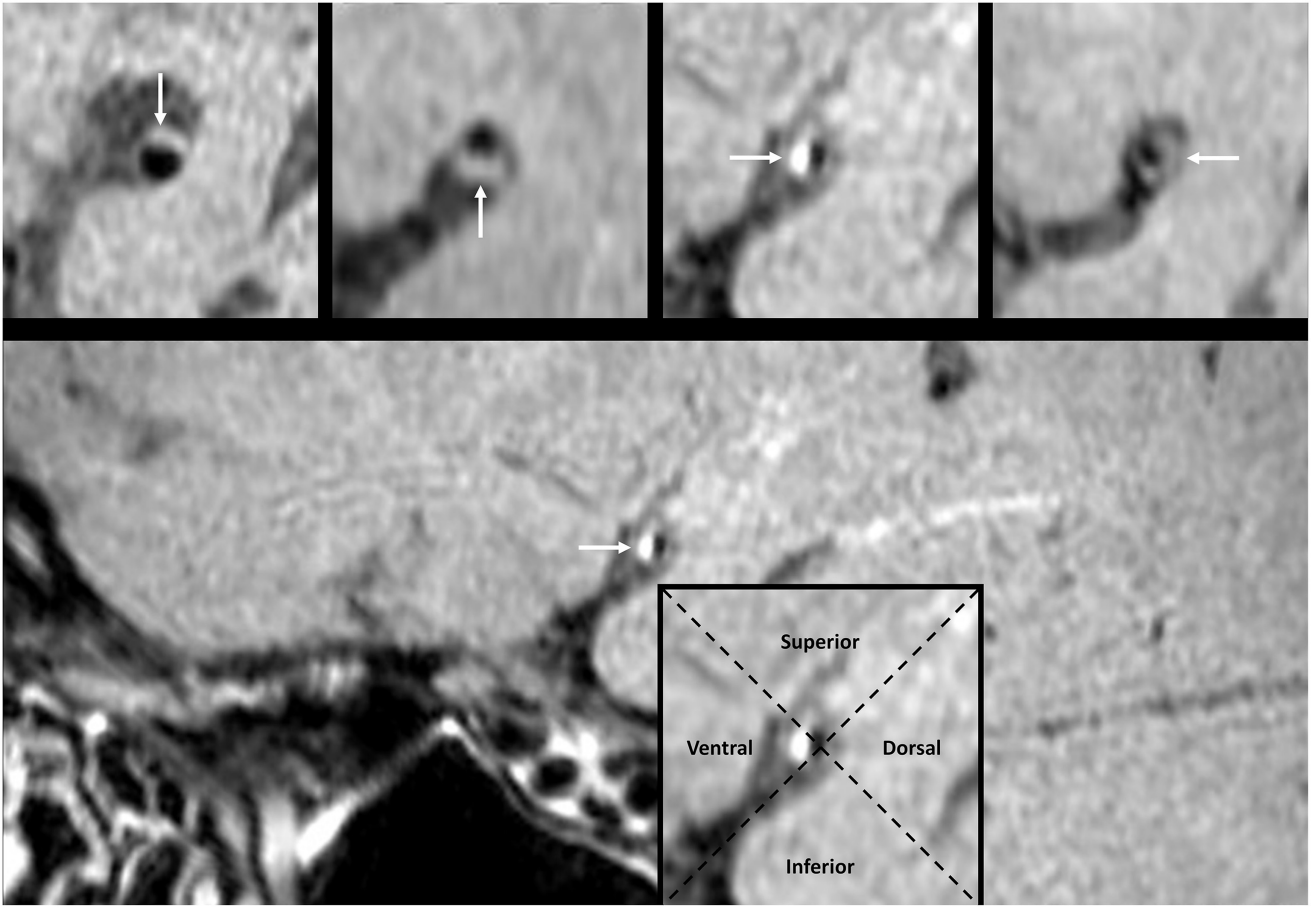
The pattern of MCA plaque wall distribution on HR-MRI. The white arrow points to the plaque in a cross-sectional image. The intersection of two dashed lines at the luminal center groups each cross-sectional image into four quadrants. The plaque in the imaging cross section may locate at the superior, inferior, ventral, or dorsal wall, respectively. HR-MRI, high-resolution magnetic resonance imaging; MCA, middle cerebral artery.

**Figure 4:**
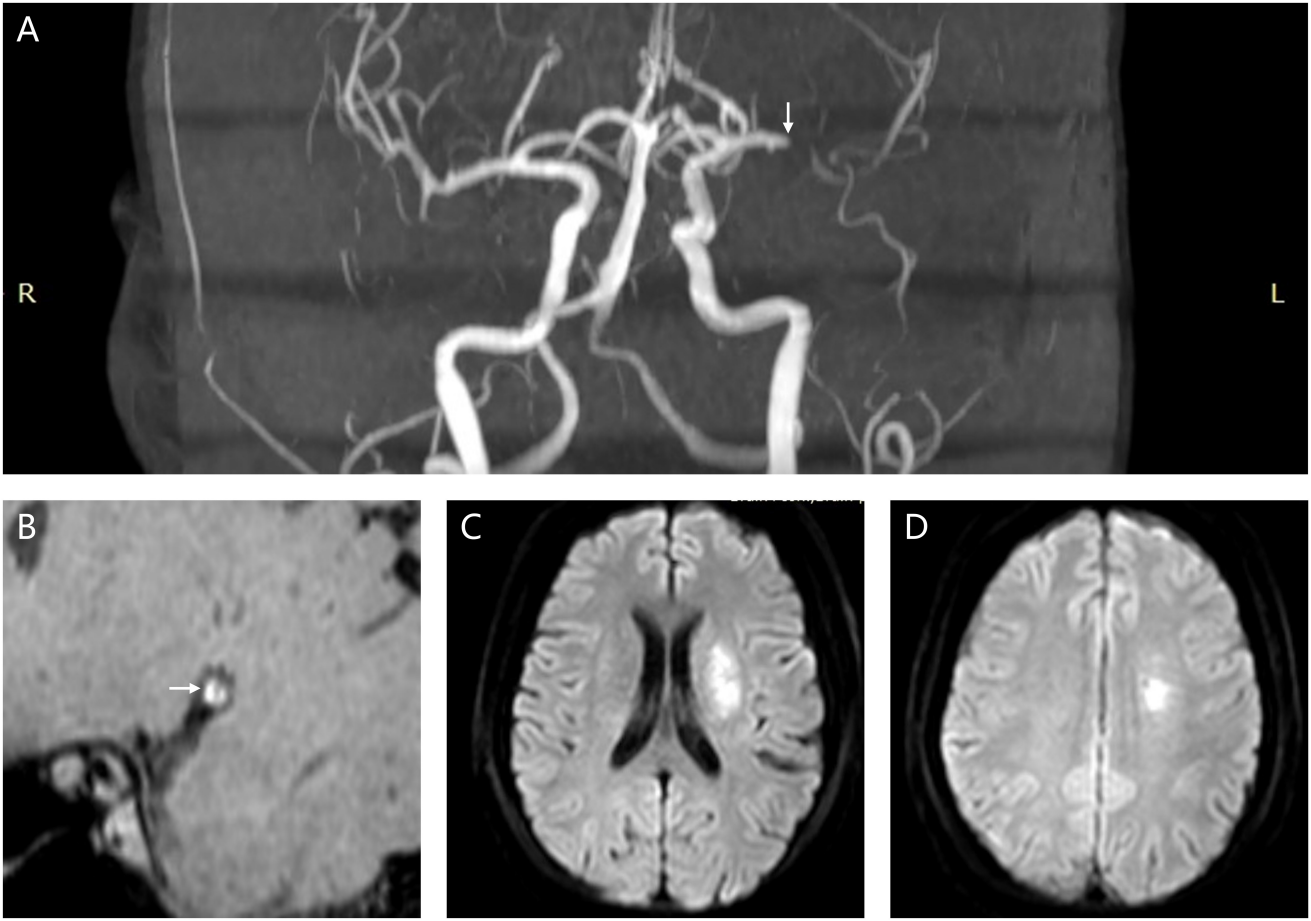
A female patient (61 years old) diagnosed as acute ischemic stroke due to atherosclerotic stenosis in the left MCA M1 segment. **(A)** The white arrow points to the symptomatic MCA atherosclerotic stenosis (MRA imaging). **(B)** The white arrow points to the symptomatic MCA plaque (high-resolution vessel wall imaging). **(C,D)** Multiple infarcts involving the perforator distribution (diffusion-weighted imaging). MCA, middle cerebral artery; MRA, magnetic resonance angiography.

A plaque in the atherosclerotic MCA M1 segment was detected, if shown as eccentric wall thickening, and the thickest site on the vessel wall was > twice the thinnest point via the visual inspection (14). All cross-sectional images of the MCA atherosclerotic plaque were categorized on the basis of the plaque wall distribution into four vascular sides: superior, inferior, ventral, and dorsal (Figure 3). Each cross-sectional image was divided into one of the four quadrants. If a plaque covered two or more quadrants, the quadrant at the thickest site was selected. Each plaque was then grouped into the asymptomatic or symptomatic lesion, according to whether this plaque could result in the downstream ischemic events within the MCA M1 segment territory (18). Perforator infarction was identified as ischemia within the intracranial small-artery territory, based on the findings from diffusion-weighted imaging (19) (Figure 4).

### Statistical Analysis

Statistical analysis was conducted by the SPSS version 25.0 (IBM, NY, USA). Variables were expressed as mean ± standard deviation (SD) or percentage frequencies, when appropriate. The individual percentage wall distribution was analyzed for each atherosclerotic plaque, and generated the mean superior, inferior, ventral, and dorsal wall distribution of the total groups (14). The mean percentages of every plaque wall distribution between groups with and without incomplete COW subtypes, and between groups with and without perforator infarcts, were compared by Mann-Whitney *U-*test. The relationship between the COW integrity and symptomatic MCA plaques leading to perforator infarctions was determined by Chi-squared test or Fisher's exact test. *P* < 0.05 was considered as statistical significance. Inter-rater reliability was tested by Cohen κ or intraclass correlation coefficient with 95% confidence intervals (CIs). A coefficient > 0.81 was regarded as excellence.

## Results

### Demographic and Clinical Parameters

Eighty seven subjects with MCA atherosclerotic plaques met the inclusion criteria in the current study. The clinical characteristics of patients were shown in Table 1. Mean age was 62.39 ± 11.64 years. Fifty three patients were male. Hypertension was the most common cerebral vascular risk factor (74.7%), followed by hyperlipidemia, diabetes, and smoking. The prevalence of IS (87.4%) as an index event was higher than that of TIA (12.6%). The incomplete P-COW was more prevalent than the incomplete A-COW in patients with MCA atherosclerosis (83.9 vs. 36.8%), while the complete COW was the least detected (8.1%).

**Table 1:** Baseline clinical parameters of patients with MCA atherosclerosis.

**Characteristics**	**Subjects (*n* = 87)**
Age (years, mean ± SD)	62.39 ± 11.64
Male/Female, *n*	53/34
Hypertension, *n* (%)	65 (74.7%)
Hyperlipidemia, *n* (%)	50 (57.5%)
Diabetes, *n* (%)	29 (33.3%)
Smoking, *n* (%)	20 (23.0%)
Index event	
Stroke, *n* (%)	76 (87.4%)
TIA, *n* (%)	11 (12.6%)
COW integrity	
Complete COW, *n* (%)	7 (8.1%)
Incomplete A-COW, *n* (%)	32 (36.8%)
Incomplete P-COW, *n* (%)z	73 (83.9%)

*COW, circle of Willis; A-COW, anterior COW; P-COW, posterior COW; MCA, middle cerebral artery; TIA, transient ischemic attack*.

### MCA Plaque Wall Distribution and the Incomplete COW

Overall, 130 lesions of the MCA M1 segments were identified as atherosclerotic in this study, including 81 asymptomatic and 49 symptomatic lesions. A total of 471 image slices on HR-MRI were used for detecting plaque wall distribution in the atherosclerotic MCA M1 segments.

The plaque wall distributions of MCA atherosclerosis between groups with and without incomplete COW subtypes were summarized in Table 2. Within all lesions of the atherosclerotic MCAs, plaques in the incomplete A-COW were distributed more commonly on the inferior wall (*P* = 0.008, Table 2), but less on the ventral wall (*P* < 0.001, Table 2). Within asymptomatic lesions of MCA atherosclerosis, the incomplete A-COW had significantly more inferior plaques (*P* = 0.043, Table 2), but fewer ventral plaques (*P* = 0.008, Table 2). Similarly, within symptomatic MCA atherosclerotic lesions, the incomplete A-COW had significantly fewer ventral plaques (*P* = 0.011, Table 2). Within all MCA atherosclerotic lesions, meanwhile, plaques in the incomplete P-COW were distributed less commonly on the inferior wall (*P* = 0.032, Table 2).

**Table 2:** Relationship between MCA plaque wall orientation and the COW completeness.

**Distribution**	**Plaques without incomplete A-COW**	**Plaques with incomplete A-COW**	***P*-value**	**Plaques without incomplete P-COW**	**Plaques with incomplete P-COW**	***P*-value**
**All lesions (130)**						
Superior wall	14.9%	15.2%	0.831	12.8%	15.5%	0.632
Inferior wall	22.9%	43.7%	**0.008**	48.8%	27.4%	**0.032**
Ventral wall	50.7%	21.2%	**0.000**	29.3%	41.5%	0.398
Dorsal wall	11.5%	19.9%	0.269	9.1%	15.6%	0.494
**Asymptomatic lesions (81)**						
Superior wall	16.4%	18.1%	0.731	16.7%	17.0%	0.890
Inferior wall	21.2%	42.2%	**0.043**	42.0%	26.4%	0.151
Ventral wall	48.1%	19.1%	**0.008**	26.1%	39.8%	0.521
Dorsal wall	14.3%	20.6%	0.565	15.2%	16.8%	0.822
**Symptomatic lesions (49)**						
Superior wall	12.4%	11.1%	0.931	7.1%	12.8%	0.564
Inferior wall	25.9%	45.8%	0.091	58.9%	29.2%	0.110
Ventral wall	55.3%	24.3%	**0.011**	34.0%	44.3%	0.585
Dorsal wall	6.4%	18.8%	0.259	0.0%	13.7%	0.149

*COW, circle of Willis; A-COW, anterior COW; P-COW, posterior COW; MCA, middle cerebral artery*.

### Symptomatic MCA Plaques, Perforator Infarctions, and the COW Completeness

To further the understanding of the clinical impact of our findings, we then investigated the association between the plaque wall distribution of symptomatic MCA atherosclerosis and perforator infarcts (Table 3), and between the COW integrity and symptomatic MCA plaques causing perforator infarcts (Table 4). As shown in Table 3, symptomatic MCA plaques within perforator infarctions were more possible to be distributed on the superior wall than those without perforator infarctions (*P* = 0.045). As presented in Table 4, however, no statistical differences were observed in the locations of symptomatic MCA plaques resulting in perforator infarctions depending on the COW structures (*P* > 0.05).

**Table 3:** Relationship between symptomatic MCA plaque wall orientation and perforator infarctions.

**Distribution**	**Plaques without perforator infarcts (*n* = 18)**	**Plaques with perforator infarcts (*n* = 31)**	***P*-value**
Superior wall	2.1%	17.6%	**0.045**
Inferior wall	32.4%	35.0%	0.972
Ventral wall	51.2%	37.7%	0.261
Dorsal wall	14.3%	9.7%	0.580

*MCA, middle cerebral artery*.

**Table 4:** Relationship between the COW completeness and symptomatic MCA plaques inducing perforator infarctions.

**COW integrity**	**Plaques without perforator infarcts (*n* = 18)**	**Plaques with perforator infarcts (*n* = 31)**	***P*-value**
A-COW			0.417
Complete, *n* (%)	12 (66.7%)	17 (54.8%)	
Incomplete, *n* (%)	6 (33.3%)	14 (45.2%)	
P-COW			0.229
Complete, *n* (%)	1 (5.6%)	7 (22.6%)	
Incomplete, *n* (%)	17 (94.4%)	24 (77.4%)	

*COW, circle of Willis; A-COW, anterior COW; P-COW, posterior COW; MCA, middle cerebral artery*.

### Inter-rater Reliability

Inter-rater reliability on the evaluation of the plaque detection and the COW integrity was excellent (for the plaque detection, coefficient = 0.892, 95% CI 0.735–0.958; for the COW integrity, coefficient = 0.886, 95% CI 0.722–0.956).

## Discussion

In the current study, first, we confirmed that the incomplete COW types commonly existed in patients with MCA atherosclerosis. Second, the distinctive feature of plaque wall distribution in the MCA M1 segments could be distinguished between complete COW and incomplete COW types by utilizing HR-MRI. We found that the MCA plaques in the incomplete A-COW located on more inferior but fewer ventral walls, compared to those in the complete A-COW. Besides, the incomplete P-COW had fewer inferior plaques in the MCAs than the complete P-COW. Third, superior plaques of symptomatic MCA atherosclerosis was significantly associated with branch occlusive disease. Our observations might gain an insight into the cerebral vascular mechanisms of MCA atherosclerosis.

The structural variations of the COW have existed in the normal populations since the initial formation of the COW (20). In a previous MRA study, an incomplete P-COW was identified in 83.9% of the healthy male individuals, an incomplete A-COW in 21.4%, and a complete COW in 12.2% (7). Other MRA studies also revealed the prevalence of each COW structural type in stroke patients (16, 17). The results indicated that 79.7% of symptomatic patients with carotid artery atherosclerosis had an incomplete P-COW, 40.7% had an incomplete A-COW, and 12.7% had a complete COW (16). These findings were comparable with our investigations into the rates of the COW subtypes (incomplete P-COW, 83.9%; incomplete A-COW, 36.8%; complete COW, 8.1%) in patients with MCA atherosclerosis. Of note, the different study subjects might cause the fluctuation in the prevalence of the COW structures.

Previous research suggested that the MCA plaques were mainly distributed on the inferior and ventral walls, and the asymptomatic MCA atherosclerotic lesions had more inferior plaque wall orientation (14). Our study further indicated that compared to the complete A-COW type, the incomplete A-COW type was positively correlated with the inferior wall plaques, but negatively with the ventral wall plaques in the atherosclerotic MCA M1 segments. This different pattern of the MCA plaque wall distribution between the complete and incomplete A-COW was presumably caused by the alteration of hemodynamic flow and wall shear stress. The plausible explanation could be given: First, the COW anatomical variations could largely affect the flow patterns within the MCAs (11, 21, 22). Second, the local changes of the hemodynamic conditions might then have a major influence on the wall shear stress spatially distributed on the MCA walls (21, 23). Particularly, the decreased levels of wall shear stress might contribute to the endothelial cell dysfunction and inflammation, ultimately accelerating the progression of atherosclerosis (24–26). Our main findings might support this hypothesis, and help to enhance our comprehension of the vascular pathogenesis of MCA atherosclerosis. This study also showed a negative relevance between the incomplete P-COW and the inferior wall plaques in the MCA M1 segment. Yet, further verification in a larger study cohort is still warranted.

In this study, we also explored the possible clinical impact of our findings. The results confirmed the prior research reporting that the superior plaques in the symptomatic MCAs were robustly related to the occurrence of branch occlusive disease via occluding the perforator orifice (14). On the other hand, we did not find any significant association of the COW integrity with symptomatic MCA plaques causing branch occlusive disease, despite our observation that the COW integrity might affect the plaque wall distribution pattern of MCA atherosclerosis. Our results are potentially meaningful in the clinical practice after all. The anatomic characteristics of the intracranial arteries were reported to impact on the outcome of endovascular therapy (27, 28). Accordingly, evaluating the COW integrity before the endovascular procedures may help to target the non-random plaque localization in the MCAs, and then improve the efficacy and safety of interventional treatment (29, 30). Follow-up studies are needed in the future to validate this potential value of assessing the COW completeness.

Limitations should be considered in the current study. First, a selection bias could be introduced in this study. Subjects with chronic IS and TIA were excluded to avoid any confounding factors, as the process of the intracranial atherosclerotic plaques might be affected by time course, medical therapy, and lifestyle changes (31). Second, the COW integrity was analyzed by TOF MRA, which showed relative insensitivity to identify low blood flow and intracranial small vessels (32). Thus, the incidence rate of patients with complete COW might be underestimated. Third, regarding the hemodynamic role of the COW integrity on the MCA plaque wall orientation, no recent studies could provide any direct evidence. Our hypothesis should be further verified by the computational fluid dynamics research.

## Conclusion

The incomplete COW types were prevalent in patients with MCA atherosclerosis. The incomplete A-COW was associated with more inferior but fewer ventral wall plaques in the atherosclerotic MCA M1 segments, while the incomplete P-COW was found in relevance to fewer inferior MCA plaques. Besides, the occurrence of branch occlusive disease was correlated with the superior wall plaques of symptomatic MCA atherosclerosis. Our observations may highlight the hemodynamic function of the incomplete COW structures on the pattern of plaque wall distribution in MCA atherosclerosis, but the clinical impact still requires further investigations.

## Data Availability Statement

The datasets generated for this study are available on request to the corresponding author.

## Ethics Statement

The studies involving human participants were reviewed and approved by the Joint Chinese University of Hong Kong-New Territories East Cluster Clinical Research Ethics Committee (The Joint CUHK-NTEC CREC). The patients/participants provided their written informed consent to participate in this study.

## Author Contributions

JL analyzed imaging data and drafted the manuscript. LZ and W-JY recruited patients. C-YS-T participated in analyzing imaging data. TL participated in study coordination and recruited patients. X-YC conceived the study, participated in its design and coordination, and revised the manuscript. All authors contributed to the article and approved the submitted version.

## Conflict of Interest

The authors declare that the research was conducted in the absence of any commercial or financial relationships that could be construed as a potential conflict of interest.
